# Everyday wishes of older people living with dementia in care planning: a qualitative study

**DOI:** 10.1186/s12913-022-07606-1

**Published:** 2022-02-12

**Authors:** Md Razib Mamun, Yoshihisa Hirakawa, KM Saif-Ur-Rahman, Tomoka Sakaguchi, Chifa Chiang, Hiroshi Yatsuya

**Affiliations:** 1 Department of Public Health and Health Systems, Graduate School of Medicine, Nagoya University, Nagoya, Japan; 2 Health Systems and Population Studies Division, icddr,b, Dhaka, Bangladesh

**Keywords:** Dementia, Everyday wishes, Activities of daily living, Older people, Qualitative research

## Abstract

**Background:**

The dementia care policy in Japan emphasizes the views of people living with dementia in care planning. An exploration of the everyday wishes of older people living with dementia can help clarify their priorities and assist in improving dementia care. This study aimed to explore the everyday wishes of older people living with dementia in Japan.

**Methods:**

This qualitative study was conducted in Aichi prefecture in Japan. Older people with mild to moderate dementia were considered for inclusion. Participants were recruited from a dementia outpatient clinic. In-depth interviews were conducted with 36 participants in the same dementia outpatient clinic from January to October 2019. Audio-recorded interviews were transcribed verbatim. Inductive content analysis was carried out to analyze the data.

**Findings:**

Participants expressed their everyday wishes within five themes (desire of being connected, freedom to decide, involvement in activities, status quo, and self-reliance). Older people living with dementia loved the connection with their family and wanted to have an enjoyable life by engaging in several activities without others’ interference. They desired to maintain the status quo and not be a burden to others.

**Conclusions:**

This study provides evidence on the everyday wishes of people living with dementia. Identified wishes are mostly on emotional aspects of their daily lives. The findings of our study might help provide care for the people living with dementia considering their wishes. Further exploration, including people with severe dementia, is needed.

**Supplementary Information:**

The online version contains supplementary material available at 10.1186/s12913-022-07606-1.

## Background

Globally, the number of older people living with dementia (PLwD) is increasing. Dementia affects about 50 million people, estimated to reach 152 million by 2050 [1]. Japan is one of the super-aged countries and faces a rapid increase in the number of PLwD. In Japan, about 7 million people are estimated to be with dementia in 2025 [2]. Considering the increasing number globally and its consequences, the World Health Organization (WHO) declared dementia a public health priority and developed a global action plan focusing on improving the physical, psychological, and social wellbeing of PLwD, their caregivers, and families [3]. Changes in emotional responses are often common in PLwD. Earlier research identified that emotional factors play a crucial role in determining wellbeing in PLwD [4–7]. Besides the health care needs, the emotional needs of PLwD and their caregiver have also been acknowledged by WHO [8]. Considering the increasing number of PLwD and their medical and emotional needs, building a comprehensive care system that fosters and maintains collaboration among physicians, nurses, psychologists, and social workers in the community has been highlighted [8]. Though personal preferences of PLwD need to be acknowledged for ensuring emotional support, caregivers often do not know the actual needs and wishes of PLwD [9].

Lord [10] developed the New Interventions for independence in Dementia Study (NIDUS), an evidence-informed theoretical model that explained the values, approaches, and strategies in dementia care. This theoretical model highlighted the consideration of the rights and dignity of PLwD in dementia care design and delivery. It also focused on developing the care strategies based on the needs of the PLwD. Another similar concept is the empowerment of PLwD. According to McConnell [11], PLwD are empowered when they are respected, have a voice and are acknowledged, are active in deciding about their life, have the opportunity to create change through access to proper resources. It has been a vital discourse in recent days. The Alzheimer Europe promotes the engagement of PLwD in research that highlights the empowerment of PLwD in Patient and Public Involvement (PPI). Although researchers have emphasized the importance of including the direct voice of PLwD in the study, still very little is explored about the preferences of PLwD [12]. The limited literature in the area suggests that the perspectives of PLwD differ from those expressed by their caregivers/families and health professionals [13–15].

A systematic review regarding the factors associated with the quality of life of PLwD reported physical (physical health, physical independence, physical and behavioral symptoms), psychological (psychological and behavioral symptoms, dignity, self-efficacy), social (relationship and communication with others), demographics (age, sex, education, marital status) and environmental factors (living settings) [16]. This systematic review considered PLwD living in community and care institutions and factors in different stages of dementia. However, in the included studies of this systematic review, most researchers explored family members’ and service providers’ perspectives instead of PLwD themselves.

The government of Japan started a “Community-based integrated care system” in 2013 that aims to support older people to live in their familiar places. A policy package called “the new orange plan” that is inspired by the “Community-based integrated care system’ has been promoted in Japan since 2015. This policy aims to support PLwD living in their familiar places as long as possible. Building a “dementia-friendly community” has been highlighted as one of the pillars of this policy [17]. In Japan, the approach to establishing dementia-friendly communities places a strong emphasis on integrating PLwD in decision-making and having a good understanding of the need of PLwD by the people. The significant challenges to achieving the goal were reported as ignored wishes of PLwD [18] since the dementia care policy of Japan emphasized the perspectives of PLwD in care planning [17]. However, the everyday wishes of PLwD are ignored because little is known about the individual everyday wishes of older PLwD.

This study explores the everyday wishes of older PLwD in Japan. The findings of this study will inform caregivers, families, and service providers how PLwD can be supported more by delivering care in line with their wishes.

## Methods

### Design and setting

In this study, we applied a qualitative methodological approach to explore the everyday wishes of PLwD. We used the consolidated criteria for reporting qualitative research (COREQ) to inform our reporting [19]. Japan is divided into 47 prefectures for administrative purposes, which is the highest administrative division after the national government. Prefectures are administered by elected governors and assemblies. On average, the distribution of older people is similar across prefectures in Japan. A dementia outpatient clinic in Aichi prefecture in central Japan was conveniently selected for the recruitment of participants. In-depth interviews with PLwD were conducted in the same outpatient clinic from January to October 2019.

### Sampling strategy and data collection

In this study, older people with mild to moderate dementia were considered for inclusion. It was determined on the clinical basis. The Clinical Dementia Rating (CDR) global scores [20] of the participants were collected by the lead researcher from the dementia outpatient clinic to confirm the stages of dementia. Corresponding global CDR scores to the dementia stages were a score of 1 for mild dementia and a score of 2 for moderate dementia. Only non-institutionalized PLwD were considered. Both Alzheimer’s and vascular dementia were included. The eligible PLwD and their family members (if necessary) were asked for their permission to be recruited for the study. Afterward, information about the aim of the study and how long the interview would take place was provided to the potential participants and their family members (if necessary). The lead researcher set up an appointment with participants for the interview after reaching an agreement. The Revised Hasegawa Dementia Scale (HDS-R) scores [21] and information about comorbidity were collected from the dementia outpatient clinic. Convenience sampling was used to recruit respondents. The sample size was determined using the data saturation model, which was defined as when new interviews provided no additional insight related to the research aim compared to data generated from earlier interviews upon consensus among the study team. Up to data saturation, we interviewed 36 participants who met the inclusion criteria. Data were collected using a pre-specified topic guide (Supplementary file 1). In this study, the wishes of PLwD were their desire to do or have something, covering both expectations and preferences. It also included the statement on any appeal. Namely, the participants were asked about their wishes in daily life. Precisely, in Japanese, they were asked about ‘nichijohseikatsu no kiboh (everyday wishes)’, with additional explanation using the following two points: ‘yusen shitai koudou (preferences for daily activities)’, and ‘nichijohseikatsu deno kitai (expectations in daily life)’. Several probing questions were asked about the place, person, circumstances, and activity to gain greater insight. Each interview lasted around 40 min in total. The lead researcher, who has extensive expertise in dementia care and qualitative research, performed the data collection. All the interviews were digitally recorded, and detailed field notes were taken by another researcher. Interviews were conducted at health care facilities as per the convenience of the respondents. Table 1 displays the characteristics of the respondents.

**Table 1:** Characteristics of participants (*n* = 36)

Variable	n
Age (years)	
70–79	4
80–89	29
≥ 90	3
Gender	
Male	6
Female	30
Revised Hasegawa Dementia Scale (HDS-R) score	
9–14	13
15–20	23
Independence degree of daily living for the demented older	
Independent	12
Need dementia help and support	24
Care service use	
Adult day service	30
None	6
Comorbidity	
Hypertension	16
Dyslipidemia	8
Osteoporosis	4
Diabetes	3
Other diseases	4
No comorbidity	13

Note: Few participants have multiple comorbidities

### Data analysis

All recorded interviews were transcribed verbatim in Japanese and then translated into English by a bilingual researcher (Japanese and English). The lead researcher checked both the Japanese and translated interviews to cross-check data fidelity. In this study, an inductive content analysis was performed [22]. The analysis started with open coding, then the codes were grouped into categories, and the data within and between categories was compared. First, researchers involved in the analysis read each transcript several times to familiarize themselves with the data pattern. Then the meaning units of texts were extracted and labeled with codes. Two researchers coded each transcript independently. In order to enhance inter-coder reliability, the study team regularly had meetings. The codes were cross-checked, and any disagreements were discussed and resolved by consensus. The codes were then categorized into subgroups based on their similarities and differences. Similar sub-categories were grouped and labeled as categories. Finally, the categories were summarized and developed themes. The study team that comprises geriatricians, epidemiologists, and qualitative researchers performed several meetings to finalize the sub-categories, categories, and themes. Disagreements were solved by discussion until consensus was reached. The final themes were approved by the study team.

### Trustworthiness

Various strategies were followed to minimize personal bias and strengthen the trustworthiness of the data, as suggested by Guba and Lincoln [23]. Analyst/investigator triangulation was achieved by involving multiple researchers in the analysis. Debriefing sessions with the study team were held on a regular basis, during which the interpretations of the codes, subcategories, categories, and themes were discussed until agreement was reached. The study team also had peer-debriefing sessions to enrich data interpretation. The participants were contacted after data analysis to confirm the interpretation. Identified themes were shared with the participants, and they commented. A final decision was made upon consensus.

### Ethical considerations

This study was approved by the Bioethics Review Committee, Graduate School of Medicine, Nagoya University, Japan (approval number 2015–0444). Before the interview, all elements of consent, the purpose of the study, and confidentiality were explained to participants and their family members who were present on the interview date. Written informed consent was obtained, and permission to be audio-recorded was taken as well. The participants were considered competent to consent by their family members and the physician of the dementia outpatient clinic. A family member was allowed to present during the interview as a companion if the participant requested it, but they were not actively involved in the interview. In other circumstances, the presence of anyone other than the participant and researchers was restricted in order to ensure privacy. The transcripts of the interviews were made anonymously.

## Findings

Most of the participants were aged between 80 and 89 years (80.5%) and female (83.3%). The HDS-R scores of the participants were ranged from 9 to 14 (36.1%) and 15–20 (63.9%). Adult day service was used by most of the respondents (83.3%). This study explored everyday wishes expressed by the PLwD. From the qualitative data analysis of interviews, 168 primary codes emerged. The study findings were organized under five themes: desire of being connected, freedom to decide, involvement in activities, status quo, and self-reliance.

### Desire of being connected

The participants described the theme ‘desire of being connected’ from two perspectives: (1) the importance of living with family (2) attachment with family and friends. The desire of being connected in everyday life was stated as the most important wish by all participants.

All the non-institutionalized PLwD who participated in this study lived with their family. Most of the participants lived with their spouses. Some participants stayed with their son/ daughter, and few had extended families where they lived with their spouses, children, and grandchildren. Some participants lived alone but moved to stay with their children after they were diagnosed with dementia. The participants talked about the importance of living with their families. Participants informed that they had people to look after them and talked about the help that family members did. They recognized the importance of this assistance in getting through everyday life. Many also stated that their everyday lives would have worsened if they were living alone. Participants acknowledged the importance of living with the family and wished to live so under any circumstances.

A strong connection with family was highlighted as the most desirable thing for their emotional happiness. Participants stated that living with the family will be more useful when they are connected with other members of the family. Presence, mutual conversation, do something together were considered as the connection. Besides family, connection with friends was considered a vital everyday wish among participants. Participants who were relocated to a new place and could not visit friends missed their friends who used to live close by. The PLwD included in our study had a fear of being alone. They often felt unhappy when they were unaccompanied. Participants reported being negatively affected emotionally when they experienced loneliness. The essence of the findings was that the participants wanted to be accompanied by their family or friends.

*“Sometimes I live alone during the day, and my son/daughter-in-law brings dinner for me in the evening. I feel good to see them, but I feel bad when they are not with me during the day”. *(Female: Age: 82)

The study findings revealed that participants enjoy seeing family members and friends around them. Subsequently, participating in any discussion on any topic with family and friends makes them happy. They also like to have outings and parties with their beloved family and friends.

*“It’s fun to spend time with friends … Talking to family members is one of my pleasures. I feel happy watching TV together.”* (Female, Age: 85)

### Freedom to decide

The majority of participants mentioned that they were not allowed to make decisions about their daily activities, such as where and with whom to visit and their participation in social activities. The majority of the participants talked about how much they used to enjoy outdoor activities like karaoke parties, sports events, and regular shopping. They felt that their lives were being dwindled because family members were restricting and controlling their activities.

*“I used to go to the stadium with my friends to watch baseball matches. I want to go there, but my wife will not let me go”.* (Male, Age: 83)

Many participants said that, in many cases, different people prevented them from having any choice or control over what they wanted to do daily. Some participants found being questioned by family members unpleasant.

“I used to exercise; I believe that I can stay healthy by doing regular exercise. But my family members tell me that I make much noise when I exercise at home, and then I quit exercise.” (Male, Age: 81)

Some also mentioned that it was difficult for them to find out what could give them pleasure because they could not do anything as per their wishes; instead, they had to follow others’ instructions.

Participants indicated a wish for more freedom in their daily lives. Having freedom meant having control over their daily schedule and activities in daily life. Many participants, for example, requested more control over having a say in when and how they participate in preferred activities, such as attending a baseball game, going shopping, or going on a day trip, even if they had to depend on others for transportation or other support. The wish to live comfortably without interference was found universal among participants. The decision-making power at home and outside about anything related to them was commonly desired.

*“I want to spend the day without others’ (family, friends, the staff at the care center) interference.”* (Male, Age: 87)

### Involvement in activities

Participants talked about the importance of being involved in any activity and stated their chosen activities. Spending a day without doing anything could be emotionally painful for many participants. PLwD participated in our study hoped to continue enjoying activities and remaining socially engaged.

*“Being lazy at home is not good, so I want to do something.”* (Female, Age: 76)

Most of the participants stated a wish to enjoy the present time. They talked about activities they wanted to carry forward for having an enjoyable time. The preferences regarding enjoyable activities differ among Participants. Most participants expressed a desire to have a daily routine and wish to involve with something pleasant regularly, like going to a daycare center, gardening, taking a walk, playing a game, visiting different places, etc. Our study revealed that a few participants enjoyed the companion of pets. It was mentioned that the amount of effort and time required to take care of the pet made them busy. Spending time with pets also helped them combat loneliness. A few respondents expressed that taking care of pets became a part of their daily routine, which they enjoyed.

*“I’m busy and happy now because I have two cats.”* (Female, Age: 87)

It was also found that some participants, on some days, did not have anything to do; however, they did not feel depressed or bored because they had pets.

*“On days when there is no day service, I walk and play with my dog.”* (Female, Age: 81)

Many participants also wanted to have the opportunity to learn new things. They believed that it would keep them physically active and give them peace. Additionally, respondents desired to be involved in activities that would improve their condition.

*“At the day service, I am doing functional training and also doing yoga. I am not bored because I have something to do.”* (Female, Age: 81)

Despite the eagerness to learn new things, a few participants mentioned their fears due to their age and condition.

*“I wanted to learn the internet at the age of 75, but I know it’s quite difficult.”* (Female, Age: 75)

### Status quo

A status quo bias is an emotional effect that makes the participants worried that something might be lost due to change. Participants focused on having everyday life run as usual. Their habits, routines, and familiar environment were crucial to them, and they wanted to keep them unchanged.

Participants expressed their wish to live their everyday lives in the same way and do similar activities as before they were diagnosed with dementia. By doing similar activities, they wish to continue living with the same sense of self as before.

*“It’s good to keep the same rhythm of life as before.”* (Male, Age: 82).

Many of the participants were against any changes in their living environment. Furthermore, it was stated difficult for them to adapt to the new environment. Several participants expressed their disappointment as they lost contact with friends and helpful staff due to the relocation of the day service center.

*“The day service I used to go to is closed, so I have to shift to a new service center. It seems too stressful to me. I like the place and staff working there.”* (Female, Age: 82)

Some participants moved to other places to live with their children after being diagnosed with dementia. Such changes made their lives emotionally painful as it was difficult for them to cope with unfamiliar places.

### Self-reliance

The idea of self-reliance was strong among the participants. Many of them stated that they would prefer to die instead of becoming troublesome. The participants also wanted to live on their own without being a burden to others.

“*I want to be myself, and I want to die without bothering others.”* (Female, Age: 85)

Participants believed they were dependent on others to do various home jobs and everyday activities, including personal care. Several respondents desired to manage everything independently as much as possible, including taking care of themselves and not asking for too much help. Many participants also considered the caregivers, especially their spouses’ current health conditions. It was difficult for the participants to receive assistance from someone in poor health. They want to do their own things of daily livings so that they do not put too much pressure on their spouses.

*“My husband is old. He is ill too. I can go to the bathroom by myself, and I am grateful that I can walk.”* (Female, Age: 86)

Participants expressed that if they become a burden on their family and require assistance in every aspect of their daily lives, that could create conflict between them and their family when they differ in their preferences for activity which will eventually force them to follow the instruction of family members. In a few cases, participants wished to be financially independent to reduce the financial burden on the family. *“I want to do something to earn some money; I want to keep money with me all* the time” (Female, Age: 75). Having the sense that they could do things correctly was also crucial to the participants.

*“I appreciate that I can look after myself even if it is the minimum.”* (Male, Age: 80)

## Discussion

This study explored the everyday wishes of PLwD in a prefecture in central Japan. The present study showed that the participants expect an enjoyable life and emotional aspects are most important to them. Participants expressed their daily life wishes in a range of elements, including a desire of being connected, involvement in activities, freedom to decide, self-reliance, and maintaining the status quo. These views expressed by the participants did not differ by the severity of dementia. As far as we know, this is the first qualitative study to explore the direct voice of PLwD regarding their daily life wishes in Japan.

The present findings mentioned by participants are related to emotional and social factors suggest that the effort to fulfill such wishes could influence their happiness and sadness. This seems inconsistent with earlier studies conducted in the USA, Spain, and Australia that identified various aspects of physical health as dominant factors of the quality of life of PLwD [24–26]. This may be due to the fact that the present study included people with only mild to moderate dementia, and none of them had frailty.

Earlier studies identified that being with family and friends led to happiness, whereas loneliness led to sadness and depression [27, 28]. Relationships with family and friends offered the opportunity for meaningful conversations and enjoyable activities. Our findings are consistent with the literature. In our study, togetherness or connectedness is highlighted as the combination of family and friends’ physical presence and involvement in several activities with them. This was expressed as the most vital need as lack of the presence and company of family members led to depression among participants.

Though the disease trajectory makes the PLwD dependent on others, two consecutive themes in our study, namely freedom to decide and self-reliance, illustrated a great sense of dignity among participants. Inability to take care of oneself and being restricted to have the freedom to decide their activities affected one’s feeling of individual respect. Previous studies conducted in Japan identified that older people, in general, preferred autonomy and maintained dignity despite their health conditions [29, 30]. In this study, participants wish to enjoy their present time. The desire for involvement in several activities and having a daily routine were highlighted in this study. Having daily routines and engaging in pleasant activities gave the participants a sense of enjoyable life. However, desired activities were individualized. A variety of desired activities were identified in this study. One scoping review identified religious activities as one of the needs of PLwD [12]. However, in our study, religious activities were absent as daily life wishes. This may be because it is considered less important in general in Japan [31]. This study highlighted the importance of understanding the wishes of the individual related to engaging in a specific activity. Different people may wish to participate in the same activity for opposite reasons. It was important to the participants that their individual preferences be met.

### Strength and limitations

The main strength of this study is the inclusion of PLwD. They directly expressed their opinion. There are a few limitations of this study. The study included only people living with mild to moderate dementia who were not institutionalized but lived with the family, and visited the dementia outpatient clinic; therefore, the findings of this study might not apply to the later stages of dementia and who live alone. PLwD who had not visited the dementia outpatient clinic during the study period were not included, which might be another limitation. Although we considered possible heterogeneities in the expression of everyday wishes because of differences in dementia rating of this much would not be a serious issue violating the study findings, it might be one of the limitations. Indeed, we did not notice significant differences in the responses about everyday wishes according to dementia ratings. In some cases, a family member was allowed as a companion during the interview as per the desire of the PLwD. Their presence might have influenced some answers; however, we did not have such an impression.

### Practice and policy implications

Our study emphasized the principal pillar of ‘the new orange plan’, the viewpoint of PLwD. In our research, participants expressed vital elements like the desire of being connected, involvement in activities, freedom to decide, self-reliance, and maintaining the status quo. Drives to establish a supportive environment and dementia-friendly community for PLwD may fall short if broader dementia contexts are not considered, such as the variations in everyday wishes. Family members, friends, and neighbors have a crucial role in achieving dementia-friendly communities. The findings from this study illustrated that most participants desired to maintain a connection with friends and do outdoor activities. Friends and neighbors of PLwD need to understand their wishes and support accordingly. Caregivers (family members, friends, and health care service providers) respect for the sense of dignity and autonomy of PLwD is crucial. There is a need for a practical solution that will mutually benefit both PLwD and their family members in care planning. However, to do so, the specification, along with priorities of wishes and how to meet their needs to be explored. Making their wishes explicit will also allow family members and health care service providers to understand when desires are inconsistent or unachievable. It will also allow them to apply alternative options. Emotional aspect focused care can be achieved through acknowledging the needs of PLwD, especially their desire of being connected with family and others, starting dementia care programs that support the access to take part in enjoyable activities, and securing their sense of dignity. Service providers might be mindful of tailoring services to the needs of the PLwD. Especially at care facilities, service providers may introduce an individual checklist to identify the wishes of PLwD that might be helpful for them in planning and providing dementia care, the effect of which should be confirmed by intervention study before implementation.

## Conclusions

In this study, we explored the everyday wishes of PLwD through their views. The participants expressed a strong wish to live an enjoyable life with dignity. They wanted to stay active and connected with family and friends. They wished for things around them to remain unchanged. Our study highlighted a greater influence of emotional aspects on their everyday wishes. Future research, including people with severe dementia, is required to understand the variations in wishes.

## Supplementary Information

**Additional file 1.** Topic guide.

## Data Availability

The data generated and analyzed during the current study are not publicly available due to privacy issues but are available from the corresponding author on reasonable request.

## Abbreviations

CDR: Clinical Dementia Rating
COREQ: Consolidated criteria for reporting qualitative research
HDS-R: Revised Hasegawa Dementia Scale
PLwD: People Living with Dementia
WHO: World Health Organization
