# Supplementary material for: Everyday wishes of older people living with dementia in care planning: a qualitative study
Source: BMC Health Serv Res. 2022 Feb 12;22:184. doi: 10.1186/s12913-022-07606-1 (PMC8840703; doi:10.1186/s12913-022-07606-1)
Supplement: Supplementary file 1 — Additional file 1. Topic guide. [file 12913_2022_7606_MOESM1_ESM.docx]

Topic guide

1. What is important to you in daily living?
2. What are your wishes/preferences/ expectations in everyday life?
3. What do you appreciate and what you don’t like in everyday life?
4. What do you like to do in everyday life?
5. Are there any special activities that you would like to keep up in daily life?
